# Ultrasound-guided *Ex-vivo* Retrieval of Mature Oocytes for Fertility Preservation During Laparoscopic Oophorectomy: A Case Report

**Published:** 2018

**Authors:** Enrique Perez de la Blanca, Maria Fuensanta Fernandez-Perez, Elena del Mar Martin-Diaz, Manuel Lozano, Marta Garcia-Sanchez, Carolina Monedero

**Affiliations:** 1- Department of Assisted Reproduction, Hospital Quironsalud Malaga, Malaga, Spain; 2- Department of Gynecology, Hospital Quironsalud Malaga, Malaga, Spain

**Keywords:** Cryopreservation, *Ex-vivo* oocyte retrieval, Fertility preservation, Laparoscopy, Ovarian neoplasms, Ovarian teratoma, Struma ovarii, Vitrification

## Abstract

**Background:**

Cryopreservation of oocytes is an efficient method of fertility preservation (FP) that can be applied in women suffering from gynecologic conditions that menace their reproductive future. Collection of oocytes becomes challenging in some scenarios, like the possibility of an ovarian cancer, the “*ex-vivo*” harvest of oocytes for FP, aspirating follicles directly from the ovarian specimen already excised by laparotomy or laparoscopy and it is an option for these cases.

**Case Presentation:**

In the present case report, the case of a patient with an adnexal mass suspected to be a recurrent teratoma was described who referred to our Assisted Reproduction Unit in Hospital Quironsalud Malaga for FP counseling. After controlled ovarian hyperstimulation, followed by laparoscopic abdominal examination and oophorectomy, an *ex-vivo* follicular aspiration for oocyte retrieval was performed on the specimen, using a standard ultrasound-guided procedure to ease and improve the process. All the follicles were aspirated and 5 metaphase II oocytes were obtained.

**Conclusion:**

This is to our knowledge, the first communication describing the *ex-vivo* ovarian aspiration of mature oocytes for FP using standard ultrasound guidance. Although this ultrasound guidance is not completely necessary, as other authors demonstrated previously, such a procedure permitted an easy and complete harvest of oocytes in a rare tumor with bizarre cystic formations, which made follicle recognition very difficult.

## Introduction

The finding of an ovarian mass during a pelvic examination is a frequent event in clinical daily practice, and the exclusion of malignancy is the main objective of its evaluation and management (1). In premenopausal women, most of such masses and cysts correspond to benign conditions. The current management of benign ovarian tumors or cysts in this population must be conservative in order to maintain their endocrine and reproductive ovarian functions (2). In those cases needing surgery, where benignity is the most probable diagnosis, the pre or intraoperative circumstances of a particular clinical case can make it difficult to follow a conservative attitude and cryopreservation of oocytes or ovarian tissue can be a possible option for fertility preservation (FP) (3). Live birth as a result of the use of embryos obtained from *in vitro* matured (IVM) (4) and *in vivo* matured oocytes (5) after a fertility-spare treatment of ovarian malignancy with uterus conservation has already been described.

Ovarian teratomata are the most frequent benign ovarian masses in the reproductive years, and they are bilateral in 10–15% of cases (6, 7). Trans-vaginal ultrasound scan is the best diagnostic modality with a fairly typical image of a cystic mass with densely echogenic tubercle projecting into the cystic lumen, as well as other echography characteristics showing sebaceous material (8). Several possible complications of teratomata (torsion, rupture, malignant transformation, and other less frequent ones), some uncommon clinical presentations (autoimmune hemolytic anemia, struma ovarii with hyperthyroidism, *etc*.) and a variety of unusual imaging findings, can make the diagnosis of teratoma an important clinical problem. Deciding whether following a more or less conservative approach having into account the aforementioned risks, the frequent bilateralism as well as a frequent incidence in young patients with fertility desire, is a challenging situation for a clinician.

Fatemi et al. in 2011 (9) were the first to report an *ex-vivo* retrieval of mature oocyte for FP during a laparotomy, aspirating ovarian follicles with a needle in a patient with a recurrent borderline ovarian tumor, as a method to avoid tumor upstaging after spillage of its content as a consequence of its puncture. The procedure had precedents, but was performed on unstimulated ovaries and followed by IVM of the oocytes (10, 11). Bocca et al. reported in the same year the harvest of oocytes aspirating ovaries excised by laparoscopy (12).

A case of FP was reported using mature oocyte vitrification after controlled ovarian hyperstimulation, in a patient with her left ovary affected by a multilocular cystic tumor suspected as a teratoma, with a previous contralateral oophorectomy, where a conservative attitude was rejected and where follicles were difficult to recognize from other cysts present in the tumor. The procedure was chosen after the study of the cases presented by Fatemi et al. (9) and Bocca et al. (12), but, for the first time in the literature to our knowledge, ultrasound-guided follicular aspiration was used instead of a blind procedure to ease and improve the harvesting of eggs in a morphologically complicated ovarian structure.

## Case Presentation

In Hospital Quironsalud Malaga, Spain, on June 2017, a 31-year-old woman was referred to the Assisted Reproduction Unit by the Gynecologic Department of our institution, for counseling about her options of FP, since an oophorectomy of her right single ovary affected by an ovarian tumor had been indicated.

The patient was nulligravida, with no couple, had never attempted a pregnancy, with regular menses since the age of 12. The patient had lost her other ovary, six years ago, after a laparoscopic oophorectomy due to a mature teratoma of 14 *cm* in diameter. Two years ago, she suffered a cervical conization after a diagnosis of “in situ” cervical carcinoma.

**Figure 1.**
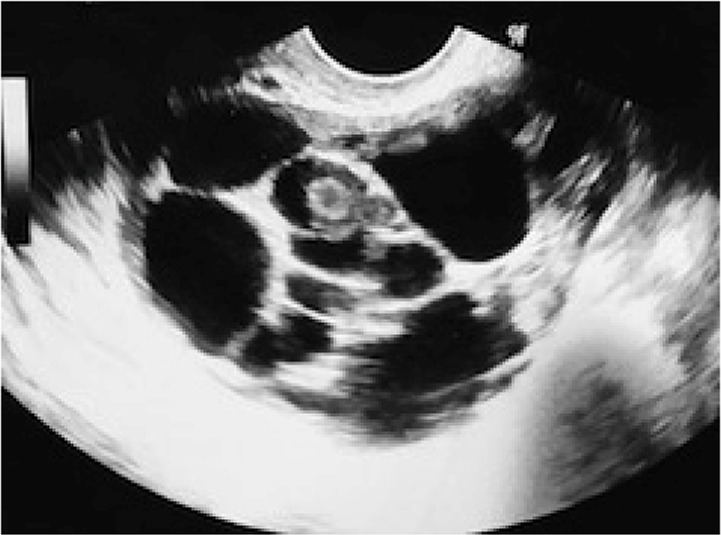
Transvaginal ultrasound image of the ovary before stimulation

During her periodical yearly reviews for her cervical process, cervical smear, pelvic examinations and ultrasounds were normal. Six months before the actual visit, she mentioned that her gynecologist described a “dense white” nodule of less than 1 *cm* that could be seen in an ultrasound in the remaining right ovary, and that it could be considered as the initiation in the development of a new teratoma, with recommendation to be observed more frequently. Only six months later, during a transvaginal scan, she presented an ovarian mass (Figure 1) of 6.5×5×4.8 *cm*, multilocular, occupying the whole ovary, with round cystic follicle-like formations, 9 to 18 *mm* in diameter, in a number of around 15, most of them anechoic. In contrast, the expected “sebaceous” content or hair, typical in teratoma could not be seen. Between the cysts, thick and hyper reflective walls could be seen, some of them with a thickness up to 1.2 *cm*. Also, an isolated densely echogenic mass of 1 *cm* in diameter, resembling the typical “dermoid plug” or Rokitansky nodule, could be observed. There was no ascitis. Another solid mass of around 2.5 *cm* in diameter was adjacent to the ovary, resembling a subserous uterine fibroid. The color Doppler mapping showed abundant vascularization in the solid central parts of the tumor. The endometrium was thin, according to her menstruation that started three days before. The rest of the pelvis was normal and the other ovary was not visible as expected.

The patient also mentioned some pelvic discomfort in the last weeks, but she had no other symptoms or weight loss in the last months. A rapidly growing teratoma was suspected. The study was completed with a magnetic resonance (MRI) that diagnosed a “probable mucinous cystadenoma” in the right ovary without visible extraovarian extension.

The choice of an oophorectomy was selected because ultrasound and MRI determined a very complex mass, with rapid growth, difficult to characterize as clearly benign, and without a significant healthy portion of the ovary to be observed in the future. Tumor markers (Ca-125, Ca 19-9, Ca 15.3, CEA) were in the normal range. Being a teratoma, the most probable type of tumor considering her antecedents, neither the ultrasound nor the MRI, could absolutely confirm the diagnosis, and the disposition of the tumor did not permit to think that a conservative surgery was possible since no “normal” areas of the ovary could be distinguished.

After a thorough discussion about all medical and personal factors (residence in Northern African zone with difficulties for medical surveillance, absence of a couple and no immediate reproductive chances), the patient accepted laparoscopic surgery involving oophorectomy and other possible actions derived from the surgical examination of her abdomen.

A proposal for oocyte cryopreservation, following ovarian stimulation and *ex-vivo* follicular aspiration was offered to the patient, after extensive information and explanation of the procedure, possibilities and risks. The aim of the extracorporeal follicular aspiration was to ease the aspiration of the follicles in such an atypical ovary and to avoid spillage of uncertain tumoral content in the abdominal cavity. Patient was informed about all the aspects of oocyte cryopreservation and blood test was performed to assess infectious status (B and C hepatitis, HIV, syphilis) and other clinical and preoperative parameters. Tests to determine anti-müllerian hormone levels and a thyroid profile were also performed. Written consent for fertility preservation was obtained according to our Institutional Review Board.

An ultrasound estimation of her ovarian reserve through antral follicle count (AFC) was not feasible due to the characteristics of the ovary, already full of follicle-like formations. With a BMI of 21, 48 *kg/m*^2^ , and an intention to obtain as many oocytes as possible, after obtaining written consent for all the procedures, and the patient being coincidentally in the fourth day of her cycle, an injection of chorifollitropin alpha 150 *mg* (Elonva, MSD) was indicated, adding a daily injection of 0.25 *mg* of ganirelix (Orgalutran, MSD) from the 6th day of stimulation. On the seventh day after the injection, some apparently new follicles could be distinguished from the other cysts with difficulty; comparing with previous images, three to four probable follicles were seen, with 13–15 *mm* in diameter. On this day, hormonal levels were: estradiol 906 *ng/ml*, progesterone 0.86 *ng/ml*, and LH 1.12 *ng/ml*. Anti-müllerian hormone levels were received this day, with a value of 1.1 *ng/ml*.

On the 8th and 9th day, 200 units of r-FSH (Gonal-F, Merck) were added to the ganirelix injection and, finally, on the 10th day, a dose of 250 *mcg* of recombinant HCG (Ovitrelle, Merck) was subcutaneously injected. The election of the moment for the trigger was based more on our clinic′s average length of antagonist protocol cycles (9 days of stimulation) than in the diameter of the follicles, since distinguishing growing follicles from tumor cysts images was somehow difficult. Thus, a new hormonal determination was considered unnecessary.

Surgical intervention was scheduled to start 35 *hr* after the HCG injection. Laparoscopy under general anesthesia started on time. Absence of ascitis or lesions in the peritoneum and abdominal organs surfaces could be observed. The left fallopian tube was absent but a small piece of ovarian albuginea, 5 *mm* in diameter, could be distinguished in the left uteroovarian ligament, with no identifiable follicular structures. No adhesions were noted in the abdominal cavity or pouch of Douglas. The right ovary was free of adhesions, easily movable, presented a lightly irregular but smooth surface and some protruyent amber-translucid cystic formations could be distinguished among some sclerotic and opaque-greysh areas. The right fallopian tube looked normal. A subserous pediculated fibroid of 25 *mm* in the posterior wall of the uterus, close to the uteroovarian right ligament, was easily removed. The patient presented a 6 *cm* scar from the previous oophorectomy in right lower quadrant area which was considered enough for a safe extraction to avoid spillage of ovarian content and rupture of the follicles. Thus, after lavage of peritoneal cavity with saline serum and collection for cytologic evaluation, oophorectomy was performed carefully without any rupture. The specimen was placed in a bag and extracted through the right lower quadrant incision without other difficulty than a little extension of its length to facilitate the extraction. Then, the specimen was taken to the assisted reproduction laboratory, contiguous to the operating room where the surgery was completed. No other suspicious or metastatic lesions were observed in the abdominal cavity. The transport of the bag was made in a stainless steel recipient 30×10 *cm*, partially filled with 500 *ml* of NaCl 0.9% at 37°*C*.

**Figure 2.**
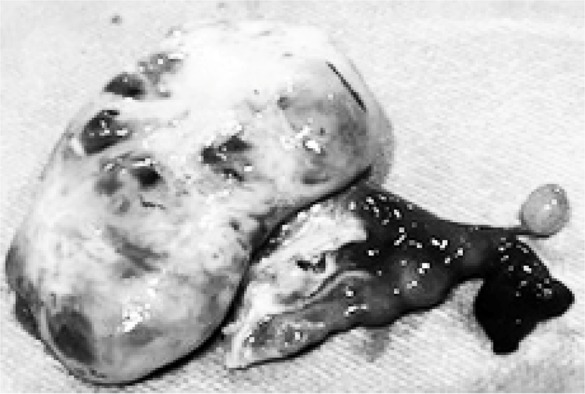
Adnexectomy specimen before follicular aspiration

Once in the laboratory, the bag was rinsed with NaCl 0.9% and its content was poured in a Petri dish, to observe if any possible follicular rupture during the extraction could have left any oocyte in it. The specimen, 8×6×5 *cm* in diameter, was placed minutes after the operative section of vascular pedicles, in another stainless steel recipient 30×10 *cm*, partially filled with warm saline and Sydney IVF Gamete Buffer (COOK Medical) (Figure 2).

**Figure 3.**
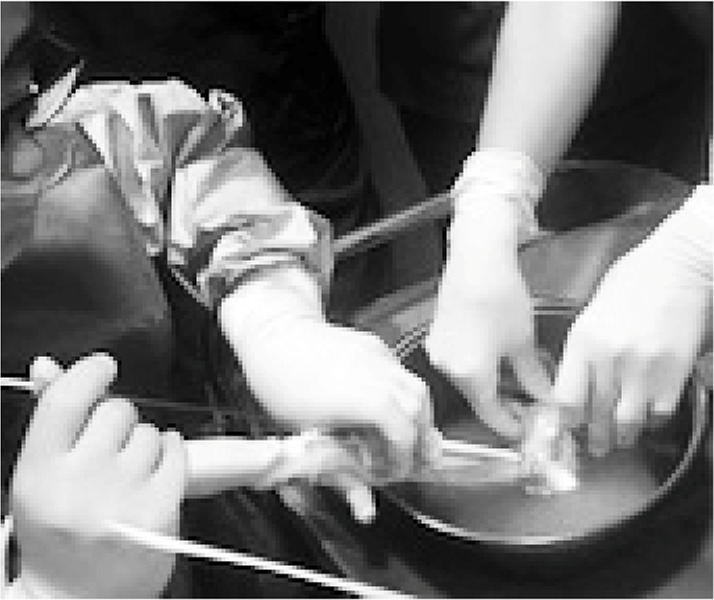
Follicular aspiration using vaginal ultrasound probe

**Figure 4.**
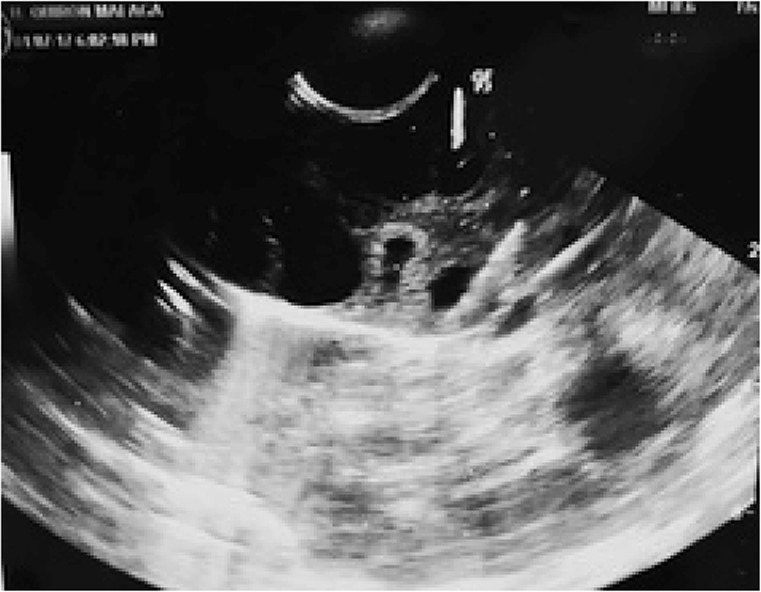
Ultrasound image during guided needle follicular aspiration

With the help of an assistant carefully holding the ovary, in a sterile way with latex and powder free gloves, follicle aspiration was performed with ultrasound guidance (ultrasound system Logic P3, vaginal probe E8c, General Electric) using the vaginal probe as normally done in conventional oocyte retrievals, covered with a latex free sterile cover and equipped with a plastic disposable guide (Figure 3). For the follicle aspiration, a single lumen needle (Ovum aspiration needle 17GA/30 *cm*, Cook Medical) was used as in normal routine vaginal procedures. The image obtained was very satisfactory, and a specular image of the specimen was formed due to the rebound of ultrasound in the bottom of the steel recipient. The aspiration at a negative pressure of 150 *mm* Hg with a follicle aspirator (ASPI-3, Labotect) resulted in traditional vaginal retrievals, and was started on those follicles which ultrasound echogenicity and size resembled most as normally observed ones in conventional IVF procedures (Figure 4). The obtained fluids were collected in six and a half, 14 *ml* tubes, placed in a heating block (Labotect) at 37°*C* and taken under the laminar flow chamber for microscopic analysis. During the aspiration, if a non clear fluid was obtained, the aspiration system was rinsed with media for the system to be clean for the next cavity to be aspirated, in order to avoid any damage on the oocyte that could be produced by any fluid different than the follicular one such as blood, mucus, sebum, *etc*. Eighteen cavities (approximately, 8 to 20 *mm* in diameter) were aspirated in total, filling 6 and a half tubes, and two of them in which content was clearly not serous fluid, were ignored. The first 7 cavities were aspirated, 3 tubes filled and they contained 5 cumulusoocyte complexes, while the other one was found in a fifth tube filled with mixed serous and bloody material. The fourth, sixth and seventh tubes did not contain any oocyte. During the filling of the fourth tube, a yellow dense fluid appeared in small amount, so the system was rinsed to continue with the fifth tube. The aspiration procedure was completed in 9 *min*.

Six cumulus-oocyte complexes were retrieved, all of them aspirated from the ovary. After 30 *min*, embryologists proceeded to denudation with hyaluronidase 80 *U/ml* (SAGE IVF Inc). Five metaphase II oocytes and one metaphase I were characterized. All mature oocytes were vitrified using Cryotop (Kitazato) and the method described by Kuwayama (13).

Postoperative period was completely normal and the patient was discharged 48 *hr* later.

The pathology study diagnosed a subserous uterine fibroid, a normal fallopian tube, and a benign struma ovarii (monodermic mature cystic teratoma) with cytologic study of peritoneal fluid negative for malignancy. Thyroid function profile tests from blood samples obtained on the day of the surgery (TSH, T4, free T4, and T3) were performed after knowing the pathologist′s report, and the results were completely normal.

## Discussion

Fortunately, the majority of ovarian masses in premenopausal women are benign. Decisions in the management of ovarian tumors are greatly influenced by the age and reproductive profile of the patient since it is desirable to preserve as much as possible their endocrine and reproductive performance.

In the present case, the clinical evolution, age, negative tumor markers and a history of oophorectomy due to a mature teratoma, suggested the possibility of a new teratoma arising in the contralateral ovary, something that occurs in 10–15% of all cases (14). Unfortunately, our patient had an earlier oophorectomy, which made decisions in her case even more difficult than usual, but every alternative was taken into account and discussed as it is currently recommended.

Pure struma ovarii is a rare tumor, comprising 3% of all ovarian teratomata (14). In the vast majority of cases, it cannot be preoperatively differentiated from mature cystic teratoma without fatty tissue, cystadenoma or cystadenocarcinoma, endometriosis, tuboovarian abscess or metastatic tumors (8).

Laparoscopic approach is the preferred way of surgical removal of presumable benign ovarian masses. It is generally recommended to do it through the umbilical port (2) causing less postoperative pain and quicker retrieval time. In the present case, as previously discussed with the patient, an extension of the lower right quadrant port over a previous operation scar was chosen.

Preservation of gametes is, when possible, the FP method of choice instead of cryopreservation of ovarian tissue. It is not known whether a recurrence of germinative cells tumor when using cryopreserved oocytes from an affected ovary is possible. In a review including 12 patients previously treated by malignant ovarian cell tumors (15) who received fertility treatment, 10 of them (83%) conceived. In three of them, an IVF was performed and all of them conceived but there are no data available about the pregnancies outcomes. A case report from Oliveira et al. (16) described the presence of a parthenogenetic embryo within a cohort of 20 oocytes obtained from a remnant ovary affected by teratoma in a patient with a previous contralateral oophorectomy for another teratoma, 4 years before. In that case, 8 normally fertilized embryos were obtained from 13 MII oocytes, and three embryos were transferred resulting in the live birth of a normal singleton healthy baby. In a series published by Caspi et al. (17), of six patients affected by ovarian teratomas who underwent IVF (four) or ovulation induction (two), five of them conceived and six healthy babies were born.

Although cryopreservation after *in vitro* maturation (IVM) of immature oocytes is a technique that has shown satisfactory results (4, 18), in some cases, cryopreservation of already mature oocytes obtained after ovarian stimulation is preferable, as it is easier and has demonstrated a higher efficiency in terms of live birth rates, specially in young patients with a good ovarian reserve (19, 20).

Usually, in nonhormone-dependent tumors, as it is the case of primary ovarian neoplasms, there is no evidence of an adverse effect of ovarian stimulation (9, 11, 21) although some authors prefer to use aromatase inhibitors to avoid high estradiol concentrations that could induce a theoretical hormone-stimulated tumor proliferation (22).

In our patient, young but with only one ovary and an impossible AFC, ovarian response was unpredictable. Anti-müllerian hormone (AMH) levels could have helped for estimation but the patient was on her fourth day of the cycle so the procedure started without AMH results. On the other side, as the cycle was not planned for fertilization and transfer, and the risk of hyperstimulation syndrome was virtually eliminated, a 150 *mcg* dose of chorifollitropin alpha was chosen, as such protocol has demonstrated good results in case of normal and suboptimal responders (23) and with the advantage of a significant lower number of injections that was thought to ease patient treatment in a delicate moment of her life.

The moment of the trigger was chosen according to the average of days at which this trigger occurred in our clinic since follicle measurements were not possible. The surgery was planned to start 35 *hr* later, thus surgeons had time to perform the oophorectomy around 36 *hr* after to maximize the chances of obtaining mature eggs with a minimal risk of spontaneous ovulation.

When surgical integrity of the ovary is preferable, “in pelvis” oocyte retrieval by means of traditional transvaginal follicular aspiration must not be the first choice. In cases where a teratoma is suspected but there is a clearly normal proportion of the ovary where follicles can develop and be aspirated (24), the risks of spillage of the content of the tumor are small, although a special effort must be made to avoid the puncture of the cysts. In our case, a tumor with a multilocular appearance resembling a hyperstimulated ovary and without a clearly “healthy” distinguishable portion of the ovary, a safe follicle transvaginal aspiration was considered impossible.

Fatemi et al. (9) were the first in publishing a case of *ex-vivo* retrieval of mature oocytes obtained from ovaries previously stimulated with gonadotropins and extirpated through laparotomy. In our case, after the laparoscopic observation of the absence of suspicious lesions in the abdominal cavity, extraction of the ovary using an endobag was decided. Bocca (12) described a similar laparoscopic procedure but using a subsequent suprapubic incision (“minilaparotomy”). In our case, the existing previous scar in right lower quadrant motivated us to use this incision, with no problems for the extraction. During the chirurgical inspection of the abdominal cavity, absence of malignancy was even more evident, but conservative treatment with a partial oophorectomy would have been impossible from a safety point of view, since the tumor occupied the ovary and was completely undistinguishable from any unsuspicious area of the gonad.

In the cases of *ex-vivo* oocyte retrieval reviewed in the literature, time of ischemia, from the oophorectomy to oocyte collection, is described in terms of less than half an hour, but not much importance is given to this issue. In animal experiences, immature oocytes can survive and be *in vitro* matured after being retrieved several hours after death (24). In some “Transport IVF programs”, oocytes are retrieved and transported in tubes to a remote laboratory during 1,5 to 3 hours with the same results in fertilization and pregnancy rates as those immediately processed (25). Considering this, in our case with a maximum interval of 28 *min* from the moment of the oophorectomy to the deposit of the oocytes under the embryologist′s microscope, timing may be considered as perfectly adequate.

According to the images observed during the ultrasound diagnosis and during the monitoring of the ovarian stimulation, a presumable difficult aspiration of the follicles was foreseen, and our team thought that it was possible to ease and improve the oocyte retrieval described by Fatemi et al. (9) using ultrasound-guided aspiration on the ovarian specimen. Coincidentally, during the days of stimulation, another advanced e-publication ahead of print about the same issue, reported by Pereira et al. (26), explained a lower than expected harvest (seven oocytes) in a case of ovarian stimulation with a favorable AFC of 14, considering the failure to obtain more oocytes as a possible consequence of the absence of the usual echosonographic guidance. Before the day of ovarian aspiration, our team made ultrasound tests using old ovarian biopsy specimens from our pathology department and a decision to try to use this method was taken since the images obtained were very good. The procedure, finally, was easy and all the cavities resembling follicles in the ovary could be aspirated without problems.

Considering all the aspects of the case, the patient, who was a candidate for egg donation after losing her ovaries, has been able to theoretically preserve certain capacity to achieve at least one live birth with her own eggs, with an estimated probability of around 41%, considering a chance of 8.2% of live birth per cryopreserved oocyte at her age (27).

## Conclusion

This is, to our knowledge, the first communication describing the *ex-vivo* aspiration using standard ultrasound guidance, of mature oocytes for fertility preservation during a laparoscopic oophorectomy of specimen after stimulation with gonadotropins. Although this ultrasound guidance is not completely necessary, as other authors demonstrated previously, such a procedure permitted an easy and complete harvest of oocytes in a rare tumor with bizarre cystic formations which made follicle recognition very difficult.
